# Construction Notes

**Published:** 1894-06-02

**Authors:** 


### CONSTRUCTION NOTES.

### Dartford Cottage Hospital.

Mr. H. M. Stanley performed the ceremony of laying the foundation-stone of the Dartford Cottage Hospital, towards the erection of which Mr. S. M. Burroughs has contributed about £1,100. The site of the hospital is on the East Hill, and the building is expected to be completed in July by Messrs. Naylor and Sons, the contractors, at a cost of £3,000, for opening in August. Although all the money has not been obtained to meet the total cost of the building, the committee are sanguine that the amount required will soon be subscribed. It is to be a two-storey building, and the officers' quarters will be in the front part. In the back will be wards to hold 16 beds, near which there will be a special ward and a convalescent room. A duty-room facing the south will have spy windows, so that the nurses may have a full view of their charges. From the administrative buildings at the front will be a covered way to the wards at the rear. Provision has been made for the erection of a mortuary at the end of the building in the event of sufficient money being raised to carry on the work.

### The New Leith Fever Hospital.

A commencement has at last been made with the erection, at East Pilton, of the new fever hospital for Leith. The hospital will comprise four ward blocks, and a start is to be made first with the one to the north-west. It is now considerably over a year since the East Pilton site was acquired, and there have been many discussions in Leith Town Council in regard to the proposed hospital. Deputations of councillors and officials visited numerous kindred institutions in England and Scotland, and the borough architect was kept busy the greater part of the year preparing various plans and estimates of cost. There are to be four ward blocks with administrative department, and all the adjuncts of a modern hospital. Eighty beds will be provided, and the estimated cost is £30,000, including £600 for drainage works, paintings, and furnishings. The site, which was purchased for £3,000, is beyond the borough boundary. It is well adapted for the purpose of a fever hospital, being completely isolated; the Leith branch of the Caledonian Railway skirts the ground to the north.

### Additions to Cork Hospital for Women.

Additions to meet the special requirements of the above hospital have just been completed, the nursing staff and the daily applicants for indoor treatment having proved quite insufficient. Mr. James F. M'Mullers, M.S.A., was appointed architect, and the works have progressed in a satisfactory manner under his superintendence, and are now practically completed. The new wing extends in a western direction, and is four storeys in height. On the ground floor the existing corridor has been extended for the full length, an old gable having been pierced with semi-circular archways on each floor. From this corridor opens the new apartments for the lady superintendent, nurses' dining-room, and other useful rooms. On the first floor the existing rooms have been slightly altered, and new private and semi private wards have been constructed. In these private wards ladies or children have comfortable rooms, tastefully furnished and decorated, and perfectly free from all intrusion. In the semi private wards the same advantages are offered. The doors from these apartments open out to the handsome, well-constructed balcony, which is one of the greatest advantages bestowed on the patients, whether young or old. Patients can be wheeled on to the balcony. The construction is of cast-iron, which has been supplied by Messrs. MacFarlane and Co , of Glasgow. Opening from the end of this corridor is the new operation theatre, removed as far as possible from the wards, and yet easily approached from the same. This is a lofty apartment with open roof, and lighted by means of Shelley's patent glazing frame overhead, and surmounted by one of Boyle's exhaust ventilators. At the far end is a finely-designed gallery for the nurses' accommodation. On the third floor nurses' quarters are supplied. A new kitchen larder, drying closet, and large nurses' recreation rooms have all been added. The entire drainage of the existing building has been overhauled with extreme care, and a series of lavatories, approached from each ward, have been provided, which are replete with all newest improvements.

